# Protein oxidation, UVA and human DNA repair

**DOI:** 10.1016/j.dnarep.2016.05.024

**Published:** 2016-08

**Authors:** Peter Karran, Reto Brem

**Affiliations:** Francis Crick Research Institute, Clare Hall Laboratory, South Mimms, Herts. EN6 3LD, UK

**Keywords:** NER, nucleotide excision repair, ROS, reactive oxygen species, UV, ultraviolet, CPD, cyclobutane pyrimidine dimer, 6:4Py:Py, 6:4 pyrimidine:pyrimidone, ^1^O_2_, singlet oxygen, 6-TG, 6-thioguanine, Protein oxidation, Reactive oxygen species, Ultraviolet radiation, Nucleotide excision repair, Skin cancer, Mutation

## Abstract

Solar UVB is carcinogenic. Nucleotide excision repair (NER) counteracts the carcinogenicity of UVB by excising potentially mutagenic UVB-induced DNA lesions. Despite this capacity for DNA repair, non-melanoma skin cancers and apparently normal sun-exposed skin contain huge numbers of mutations that are mostly attributable to unrepaired UVB-induced DNA lesions. UVA is about 20-times more abundant than UVB in incident sunlight. It does cause some DNA damage but this does not fully account for its biological impact. The effects of solar UVA are mediated by its interactions with cellular photosensitizers that generate reactive oxygen species (ROS) and induce oxidative stress. The proteome is a significant target for damage by UVA-induced ROS. In cultured human cells, UVA-induced oxidation of DNA repair proteins inhibits DNA repair. This article addresses the possible role of oxidative stress and protein oxidation in determining DNA repair efficiency – with particular reference to NER and skin cancer risk.

## 1 Introduction

### 1.1 UV and DNA damage

Although exposure to sunlight ultraviolet (UV) radiation is beneficial and enhances vitamin D levels [1], it also causes cancer. The solar UV radiation spectrum (wavelengths 100–400 nm) comprises UVC (<280 nm), UVB (280–320 nm) and UVA (320–400 nm). Early action spectra (reviewed in [2]) identified 265 nm in the UVC region as the most biologically effective wavelength in microorganisms and identified DNA as the principal damage target. As a consequence, most research into the biological effects of UV has been based on germicidal mercury lamps emitting predominantly UVC radiation. Germicidal UV produces DNA cyclobutane pyrimidine dimers (CPDs) and pyrimidine:pyrimidone 6:4 photoproducts (6:4 Py:Pys) as predominant photolesions. The involvement of these bipyrimidine photoproducts in UV-induced mutagenesis is well established and many of the processes that underlie the biological effects of shorter wavelength UV are well understood. Their processing by DNA repair/tolerance pathways and the DNA damage responses that they elicit in both simple and more complex organisms are known in some detail [3]. Importantly, these studies have also established the essentially conserved nature of the nucleotide excision repair (NER) system that protects against cell death and mutation by removing CPDs and 6:4 Py:Pys. If unrepaired, the DNA lesions induced by short wavelength UV induce characteristic C > T transition mutations at dipyrimidine sites. These mutations are regarded as the signatures of UV exposure. Of particular relevance to this review, the seminal observations that NER is defective in fibroblasts from sun-sensitive and skin cancer-prone xeroderma pigmentosum (XP) individuals defined the relationship between unrepaired UV-induced DNA damage and skin cancer susceptibility [4], [5]. These were crucial in establishing the paradigm of an inverse relationship between DNA repair proficiency and cancer risk.

The stratospheric ozone layer filters solar emissions and the sunlight UV radiation to which we are exposed comprises approximately 5% UVB and 95% UVA. It does not contain UVC. Germicidal UV is nevertheless a reasonable surrogate for some of the effects of sunlight on skin, including mutagenesis and carcinogenesis, because CPDs and 6:4 Py:Pys are also the predominant photoproducts of the shorter wavelengths of solar UVB. At longer wavelengths, the contribution of the canonical UV DNA photoproducts to cellular damage declines sharply. Biologically relevant UVA doses induce several orders of magnitude fewer CPDs (and effectively no 6:4 Py:Pys) [6]. Unlike UVC and UVB, UVA interacts with cellular photosensitizers to generate reactive oxygen species (ROS) and ROS-mediated oxidative damage to DNA and to non-DNA targets becomes increasingly significant. Overall, however, UVA induces little DNA damage [7]. Because of this and because mutation analyses clearly identify the canonical UVB photoproducts as the main mutation-inducing DNA lesions in skin cancer development, the potential contribution of UVA to cancer risk has received less attention. This review examines the evidence that the UVA in incident sunlight influences mutation and skin cancer risk and that it does this independently of its ability to damage DNA.

### 1.2 **DNA damage and the cellular effects of UVA**

CPDs and 6:4 Py:Pys are generated in oxygen-independent reactions whereas many of the biological effects of the longer UVA wavelengths are dependent on oxygen [8]. Although UVA does cause oxidative DNA damage, extensive analysis of DNA from UVA-irradiated cells indicates that oxidative DNA lesions are likely to contribute little to its toxicity and mutagenicity. Surprisingly, CPDs (predominantly T <> T) are quantitatively the major UVA photoproduct and the contribution from oxidative DNA lesions is considerably more modest [7]. This is consistent with the relative infrequency of the signature mutation of the most common oxidized DNA base – the G > T transversions associated with DNA 8-oxoGuanine – in most UVA mutation spectra [9]. Rather, these are dominated by signature dipyrimidine C > T mutations.

The canonical UV photoproducts appear not to be significant contributors to the biological effects of UVA. NER-defective XP cells that are extremely sensitive to UVC and UVB are not hypersensitive to UVA [10]. Moreover, although the effects of UVC and UVB in excision-proficient cells correlate well with the induction of canonical UV lesions, this type of damage cannot fully explain the toxicity and mutagenicity of UVA. Thus, a CPD induced by UVC or UVB is associated with 5–10 times less toxicity and mutagenicity than the same lesion induced by UVA [11], [12]. Additionally, CPDs induced by UVA in NER-proficient cells persist for longer than the same lesions induced by UVC or UVB [13], [14], suggesting that their repair might be compromised at longer wavelengths.

Taken together, the response of cells to UVA radiation and thorough analyses of UVA-damaged DNA suggest that damage to targets other than DNA is likely to be a significant contributor to the biological effects of UVA.

### 1.3 DNA damage by UVA photosensitizers

The oxygen dependence of the effects of UVA reflects the activation of endogenous photosensitizers to generate ROS. These cellular photosensitizers have not been fully characterized but candidates include porphyrins, flavins [15] and melanin [16]. Their properties can, however, be mimicked to a certain degree by photosensitizing drugs such as the thiopurines and the fluoroquinolone antibiotics. Photosensitizing drugs are linked to an increased skin cancer risk [17], [18]. Because they amplify the effects of endogenous photosensitizers and the consequences of their interactions with UVA can be examined under laboratory conditions, these therapeutic photosensitizers offer an important approach to understanding the events associated with UVA photosensitization.

**Fig. 1:**
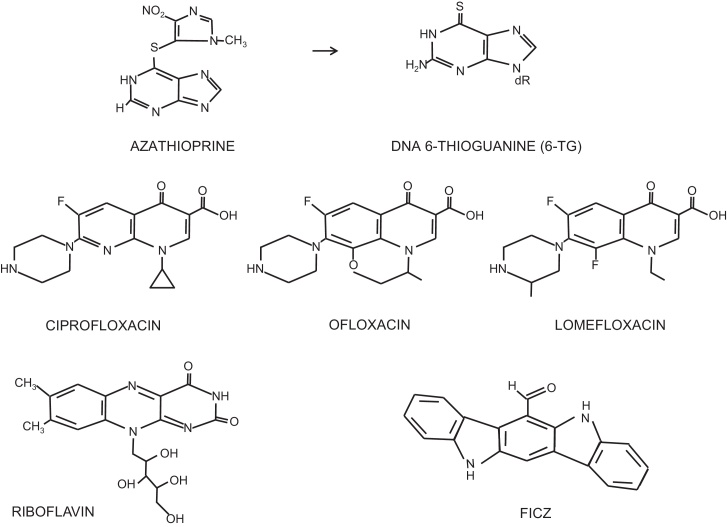
UVA photosensitizers.

The thiopurine prodrug azathioprine (Fig. 1) provides a dramatic example of an increased skin cancer risk associated with UVA exposure. An immunosuppressant that has been widely prescribed to prevent rejection in organ transplant patients, azathioprine is also used in the management of inflammatory bowel conditions. In both treatment contexts, azathioprine is associated with a significantly increased risk of non-melanoma skin cancer, principally squamous cell carcinomas (SCC) that appear almost exclusively on sunlight-exposed parts of the body. The frequency of SCC in organ transplant recipients is around 100-fold higher than normal [19] and there is a more modest but still significant increase in patients treated for inflammatory bowel disorders [20], [21]. Azathioprine treatment is associated with skin photosensitivity to UVA (but not UVB) [22]. Population studies of French and British organ transplant patients implicate solar UVA in their increased skin cancer risk. Facial tumors in French patients arise predominantly on the left side, those in British patients on the right side of the face. This is consistent with the involvement of UVA from glass-filtered UV (UVB does not penetrate glass whereas UVA does) during driving [23].

The azathioprine metabolite 6-thioguanine (6-TG) replaces a small fraction of the patients’ DNA guanine [24]. 6-TG is a strong UVA chromophore and its presence in patients’ DNA is consistent with their selective UVA photosensitivity [22]. In cultured cells, DNA 6-TG acts as both a Type I and a Type II UVA photosensitizer and interacts with UVA to generate ROS, principally singlet oxygen (^1^O_2_) in a Type II photosensitized reaction. These ROS cause widespread DNA damage including oxidized bases [22], DNA breaks [25], DNA interstrand crosslinks [26], and DNA-protein crosslinks [27]. Importantly, the DNA 6-TG/UVA combination also causes widespread protein oxidation [28].

The expectation that a contribution of azathioprine/UVA-induced DNA lesions to skin cancer development would be evident from novel signature mutations in transplant-related skin tumors was not fulfilled. The mutational spectra of the frequently mutated *PTCH* [29] and *TP53* genes [30] from skin tumors of immunosuppressed patients were found to be closely similar to those of immunocompetent individuals and were dominated by the characteristic dipyrimidine C > T mutations of canonical UV DNA lesions. These observations firmly identified UVB as the mutagen. The absence of new signature mutations in tumors from immunosuppressed individuals suggested that rather than influencing cancer risk by inducing novel mutagenic DNA lesions, the combination of sun exposure and azathioprine enhances the mutagenicity of solar UVB, potentially through damage to non-DNA targets such as proteins.

Clinical photosensitivity similar to that caused by azathioprine is a common side effect of drug treatment and is often associated with an increased skin cancer risk [17], [18]. The fluoroquinolone antibiotics are recognised clinical photosensitizers that are also UVA sensitizers in cultured cells [31]. Examples include the widely-prescribed ciprofloxacin, ofloxacin and lomefloxacin (Fig. 1). Unlike 6-TG, fluoroquinolones are not incorporated into DNA but they nevertheless replicate many of the photochemical effects of 6-TG. They are all Type II UVA photosensitizers [31] that exhibit a synergistic lethality and mutagenicity [32], [33] with UVA in cultured cells. Some, but not all fluoroquinolone/UVA combinations induce T <> T (but not other) CPDs by a triplet energy transfer mechanism [34]. Because they are a source of ROS, they all generate oxidative DNA lesions and also damage other cellular components, including proteins [33].

Riboflavin (Fig. 1) is another recognised UVA photosensitizer that generates ROS and oxidative DNA damage. Although mutations induced by riboflavin/UVA in treated cultured cells are consistent with unrepaired oxidative DNA lesions [35], some of the effects of this combination occur independently of DNA damage. Thus, DNA replication arrest induced by riboflavin/UVA is independent of the canonical ATR-, ATM or p38-dependent cell cycle checkpoints that are triggered by the presence of DNA lesions [36]. Like azathioprine and the fluoroquinolones, ROS generated by the interaction between UVA and riboflavin cause widespread damage to proteins [37].

6-formylindolo[3′2-*b*]carbazole (FICZ) is a naturally-occurring UVA chromophore. FICZ (Fig. 1), a UVB photoproduct of tryptophan, is synergistically lethal with UVA [38]. UVA activation of nanomolar concentrations of FICZ generates ROS, including ^1^O_2_ that causes oxidative damage to both DNA [38] and proteins (RB, unpublished).

The unifying feature of these photosensitizers is that their activation by UVA generates ROS that causes both DNA and protein damage.

### 1.4 Protein damage & DNA repair

Some highly resistant organisms withstand huge doses of radiation that cause massive DNA damage. The bacterium *Deinococcus radiodurans* can reconstitute functional chromosomes from tiny DNA fragments – a feat of DNA repair beyond the capability of more normally radiation-sensitive bacteria (reviewed in [39]). The astonishing DNA repair capacity of *D. radiodurans* is shared by related bacteria and certain highly radiation-resistant microscopic aquatic rotifers [40] and tadigrades [41]. It reflects particularly efficient antioxidant defences that allow these organisms to withstand the severe oxidative stress that accompanies desiccation. In particular, a multitude of very efficient antioxidant systems prevent protein oxidation and protect essential survival systems, including DNA repair [42]. The radiation resistance of these organisms highlights the susceptibility of the proteome to inactivation by oxidation and emphasizes the need to protect DNA repair proteins from damage in order to preserve genome stability.

Oxidative stress, an unwanted excess of ROS is also a threat to the viability of human cells and they invest considerable resources in anti-oxidant defences. Despite this protection, the human proteome is vulnerable to damage and inactivation by ROS if redox homeostasis is perturbed. As a source of ROS *via* its interaction with cellular chromophores, UVA radiation causes extensive protein modification [43]. In principle, all amino acid side chains can be oxidized to generate protein carbonyl groups. The sulphur groups of methionine and cysteine are also particularly susceptible to oxidation. Several DNA replication/repair proteins, including the PCNA DNA clamp and the MCM2 replication initiation factor have been shown to be targets for damage by UVA. UVA in combination with exogenous photosensitizers causes extensive ROS-dependent intersubunit crosslinking of the PCNA, Ku and RPA DNA repair complexes [33], [44], [45].

### 1.5 UVB/UVA interactions

There are indications that UVA can enhance the effects of UVB on skin. Photoaugmentation – the intensifying effect of wavelengths >320 nm on erythema induction by simulated solar radiation (SSR) was described almost 50 years ago [46]. Erythema is the skin redness associated with sunburn and is generally considered to reflect damage to and death of keratinocytes. Although subsequent investigations questioned whether these effects on erythema were truly more than additive [47], more recent studies provide support for synergy between UVA and UVB. In particular, UVA has been shown to enhance UVB-induced suppression of immune responses such as contact hypersensitivity to nickel [48]. Interactions between UVB and UVA are also apparent at the cellular level and non-toxic doses of UVA sensitize bacteria and cultured human cells to killing by UVB [49], [50]. Direct evidence that UVA can influence DNA repair was provided by the observation that UVA (365 nm) inhibits the removal by NER of UVC- (254 nm) induced photoproducts in *Escherichia coli* [49]. There is longstanding indirect evidence that UVA may also compromise NER in human cells. Thus, irradiation at 365 nm causes a dose-dependent reduction in UVC-induced unscheduled DNA synthesis (UDS, a measure of a late NER step) [51], [52]. A UVA/UVB mixture was found to be not only less effective than UVC in stimulating UDS but to actually inhibit UDS induction by both UVC and UVB [53]. Although these observations do not exclude other interpretations, they are nevertheless consistent with attenuation of NER by UVA.

More recent experiments provide direct evidence that UVA can compromise DNA repair in human cells and that it does this by damaging DNA repair proteins. UVA combined with 6-TG [28] or a fluoroquinolone photosensitizer [33] inhibits DNA repair in cultured human cells. In both cases, inhibition is the result of protein oxidation. Oxidative damage to the Ku complex impairs Non-Homologous DNA End Joining (NHEJ) [28]. The base excision repair of DNA 8-oxoG is also affected as both the OGG-1 and MUTYH DNA glycosylases that cooperate in the repair DNA 8-oxoGuanine are partially inactivated [28]. Of particular significance, oxidation of RPA compromises NER and the removal of UVC or UVB-generated 6-4 Py:Pys is impaired in cells treated with a photosensitizer and UVA [45]. These and other examples of oxidation-susceptible DNA repair proteins are listed in Table 1.

The extensive protein damage induced by photosensitized UVA [28], [33] has even found a therapeutic application. Collagen fibers are deliberately targeted for crosslinking by riboflavin/UVA in the treatment of keratoconus, a condition that weakens the cornea [54].

Importantly, these photosensitizers simply exacerbate the effects of UVA and at higher doses, UVA alone induces sufficient protein oxidation to inhibit DNA repair [55]. The formation of ROS is the key factor in DNA repair inhibition by UVA and exogenous photosensitizers that are weak sources of ROS such as the halogenated thiopyrimidines, do not significantly inhibit repair [56], [57].

These findings emphasize the vulnerability of human DNA repair to inhibition by protein damage by ROS induced by UVA either alone or in combination with an exogenous photosensitizer.

### 1.6 Oxidative stress compromises NER

If ROS induced by UVA cause protein damage that inhibits DNA repair, it is appropriate to ask whether NER is vulnerable to other interventions that induce oxidative stress. The excess ROS that define oxidative stress results from an imbalance between their formation and removal. This can be induced experimentally by compromising cellular antioxidant defences. Treatment with the glutamylcysteine synthetase inhibitor buthionine sulfoximine (BSO) depletes cells of the important antioxidant glutathione (GSH). The oxidative stress associated with GSH depletion results in an increased steady-state level of protein oxidation and BSO treatment enhances UVA-induced protein oxidation in human keratinocytes. The increased levels of protein oxidation are associated with an inhibition of NER and cells treated with UVA and BSO do not excise UVB-induced photoproducts [55]. The observed NER inhibition provides an explanation for the reversal of resistance to the anticancer drug cisplatin that follows BSO treatment [58]. By removing potentially toxic cisplatin-DNA lesions, NER is an acknowledged contributor to cisplatin resistance [59] and its inhibition would be consistent with increased cisplatin sensitivity.

Pharmacologically active, high concentrations of ascorbate (vitamin C) sensitize cells to carboplatin, a cisplatin analog. At these high concentrations, ascorbate acts in a pro-oxidant fashion to induce oxidative stress. It generates H_2_O_2_ and causes oxidative DNA damage [60]. H_2_O_2_ itself is widely used to induce oxidative stress. Although rather unreactive, it is converted into the much more reactive hydroxyl radical (OH•) by the Fenton reaction. In addition to DNA damage, H_2_O_2_ also causes extensive protein oxidation and treatment of cultured human HaCaT keratinocytes with either H_2_O_2_ or high concentrations of ascorbate inhibits NER [55].

Cells adapt to oxidative stress by diverting glucose metabolism from glycolysis to the pentose phosphate pathway (PPP). One outcome of this metabolic switch is a boost in the production of the NADPH that is required to maintain antioxidant defences by recycling oxidized GSH. By preserving reducing power, increased flux through the PPP protects against both DNA and protein oxidation. Silencing of glucose-6-phosphate dehydrogenase (G6PD), the first and rate-limiting step of the PPP, prevents this metabolic switch. G6PD silencing is associated with decreased NADPH levels, increased protein oxidation and inhibition of NER [55] . In a related outcome, compromised G6PD activity is associated with oxidative damage to the Ku protein complex, inhibition of DNA break repair by the Non-Homologous End Joining pathway and enhanced ionizing radiation sensitivity [61], [62], [63].

The TCA cycle also supplies NADPH, in this case *via* the oxidative decarboxylation of isocitrate to α-ketoglutarate by isocitrate dehydrogenase (IDH). Heterozygous mutations in the IDH1 isoform are particularly common in gliomas, chondrosarcomas and some leukemias. Gain of function IDH1 mutations confer the ability to reduce α-ketoglutarate to α-hydroxyglutarate in a reaction that consumes NADPH [64]. Mutant IDH1 is associated with decreased NADPH levels, a better prognosis and superior response to therapy. It is possible that diminished DNA repair contributes to this phenotype. In support of this possibility, restoration of high NADPH levels by inhibiting the mutant IDH1 isoform in HT1080 chondrosarcoma cells protects them against UVA-mediated NER inhibition [55].

The therapeutic effectiveness of many anticancer agents is compromised by the removal of potentially lethal DNA lesions by NER and they are more effective against tumors in which NER is compromised. This is particularly evident with the platinum-based drugs and the spectacular success of cisplatin in treating testicular carcinomas is partly a reflection of their relatively low NER efficiency [59]. It is noteworthy that oxidative stress generally enhances susceptibility to drugs that kill by damaging DNA. BSO treatment reverses the cisplatin resistance of glioma cells in culture and in xenografts [65]. Very high doses of ascorbate sensitize ovarian carcinoma cells to carboplatin [60]. G6PD overexpression is associated with cisplatin resistance [66] and G6PD silencing confers cisplatin sensitivity in cultured human cells [55]. IDH1 mutations also confer sensitivity to a number of therapeutic drugs including cisplatin [67], [68] and overexpression of wild-type IDH1 in cultured cells protects against UVB-induced apoptosis [69]. All of these observations are consistent with an association between oxidative stress and attenuated NER and a likely molecular mechanism for this NER inhibition is *via* increased protein oxidation.

In summary, DNA repair proteins are susceptible to damage and inactivation by oxidation. Interventions that are known to induce oxidative stress damage DNA repair proteins and inhibit DNA repair. UVA inhibits NER [55] and its effects are amplified by exogenous photosensitizers that increase oxidative stress. Since UVA comprises around 95% of incident solar UV and is a source of ROS *via* its interaction with cellular photosensitizers, it seems pertinent to question the extent to which solar UVA affects the NER of UVB-induced DNA lesions in skin. How efficient is NER in sun-exposed skin? How good is the protection NER provides against mutation by solar UVB? DNA sequencing data from skin tumors suggests that the effectiveness of NER might be less than optimal.

### 1.7 UVA and skin cancer mutations

**Fig. 2:**
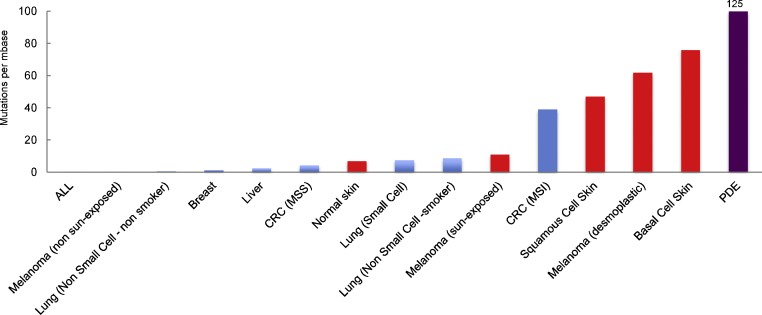
Mutation burden in human tumors. The median values for mutation frequencies in human tumors. Data are taken from refs. [91] and [92]. MSS microsatellite stable, MSI microsatellite unstable. Frequencies for melanoma also include those from refs. [93], [94] and [95] (desmoplastic). Basal cell skin carcinoma numbers are from ref. [96]. Squamous cell carcinoma frequencies include those of refs. [97] and [98]. Normal skin values from ref. [71]. PDE is colorectal carcinomas with DNA polymerase ∂/ε proofreading mutations. Mutation frequency data are from ref. [99]. Where more than one study has been reported, approximate mean values are presented. Skin and skin tumors are shown in red.

A remarkable characteristic of tumors from sun-exposed skin is their high number of mutations. The mutational loads in BCCs and SCCs are more than an order of magnitude higher than those in tumors of other anatomical sites (Fig. 2). Even morphologically normal skin [70], [71] has accumulated more mutations than many non-skin tumors. Skin carcinoma mutation frequencies are comparable to those in colorectal tumors with a mismatch repair deficiency (Lynch Syndrome) or defective replication proofreading [72] both of which confer extremely high spontaneous mutation rates. There are, however, no known DNA repair or replication error-correcting defects in skin tumors and mutations in NER genes are infrequent in skin cancer. Despite protection by NER, the overwhelming majority of mutations in skin tumors and normal skin bear the signature of solar UVB.

These huge mutational loads might simply reflect chronic sun exposure and the extremely powerful mutagenicity of UVB. In a different context, however, UVB is not a particularly potent carcinogen. UVB phototherapy is an effective treatment option for the management of chronic relapsing skin conditions such as psoriasis. It involves repeated (up to several hundred) and escalating doses of narrowband UVB that produce levels of DNA damage roughly equivalent to those induced by two minimal erythema doses (MEDs) of solar radiation [73], [74]. MED is the dose of radiation that causes just perceptible erythema on skin not normally exposed to the sun. For comparison, this means that CPD induction by each UVB treatment is approximately equivalent to 0.2 J/m^2^ UVC delivered to cultured cells. Despite this extensive UVB exposure, UVB phototherapy is not associated with a detectable cancer risk [75], [76]. In contrast, management of the same skin conditions by multiple treatments with psoralen/UVA (PUVA) which causes a different kind of DNA damage, carries a well-established and significant cancer risk [75]. The absence of detectable carcinogenicity associated with therapeutic UVB suggests that it might be less of a hazard than solar UV. We suggest that sunlight UVA contributes to this differential carcinogenicity and that by decreasing the efficiency of NER, UVA increases the mutagenicity of coincident UVB.

Skin cancer is common and available mutation data clearly implicate UVB photoproducts in its development. NER does protect against skin cancer and the extreme skin cancer susceptibility of NER-defective XP individuals testifies to the carcinogenicity of unrepaired UV-induced DNA damage. The topical application of liposomally-encapsulated DNA repair enzymes [77], [78] that ameliorates many of the effects of UV-induced DNA damage in the skin of XP patients [79], also protects repair-competent individuals [77], [78]. This indicates that the efficiency of photoproduct removal can be improved over that provided by basal NER. Estimates of the rates of photoproduct excision from the skin of UV-irradiated volunteers provide some support for this possibility. Although these vary widely and there is significant inter-individual variation that may be partly dependent on skin type (for example: [74], [80], [81]) these studies suggest that damage excision rates are generally considerably lower in intact skin than those we normally measure in laboratory experiments with cultured cells exposed to UVC. A significant fraction (>60%) of CPDs induced in skin by solar UV may persist for two or more days after irradiation.

Most of us are and will remain unaffected by skin cancer. If oxidative protein damage by solar UVA does compromise NER and contribute to mutation and skin cancer risk, its effect is likely to be subtle and cumulative. In practical terms, the possibility that the long and short wavelengths of solar UV interact synergistically in cancer development dictates that truly effective skin cancer prevention requires protection against the whole solar spectrum with efficient screening of both UVA and UVB radiation.

## Conflicts of interest

The authors declare no conflicts of interest.

## Figures and Tables

**Table 1:** DNA repair proteins known to be oxidation targets.

Protein	Reference
PCNA	Montaner et al. [44]
Ku	Gueranger et al. [28]
RPA	Guven et al. [45]; Wang et al. [82]
XRCC3	Girard et al. [83]
OGG-1	Bravard et al. [84]; Morreall et al. [85]
PARP-1	Ding et al. [86]
XPA	Grosskopf et al. [87]; Zhou et al. [88]
TFIIH(p44)	Fribourg et al. [89]
XPE	Grosskopf et al. [87]
APE1	Kelley et al. [90]
